# Fish Oil Ameliorates High-Fat Diet Induced Male Mouse Reproductive Dysfunction via Modifying the Rhythmic Expression of Testosterone Synthesis Related Genes

**DOI:** 10.3390/ijms19051325

**Published:** 2018-04-29

**Authors:** Hualin Wang, Yazheng Cai, Yang Shao, Xifeng Zhang, Na Li, Hongyu Zhang, Zhiguo Liu

**Affiliations:** School of Biology and Pharmaceutical Engineering, Wuhan Polytechnic University, Wuhan 30023, China; wanghualin313@163.com (H.W.); cyz1412779711@163.com (Y.C.); syang723@163.com (Y.S.); zhangxf9465@163.com (X.Z.); 13469956459@163.com (N.L.); zhy200422109@163.com (H.Z.)

**Keywords:** high-fat diet, fish oil, reproduction function, circadian rhythms

## Abstract

The present study aims to investigate the protective effects of ω-3 polyunsaturated fatty acids (ω-3PUFAs) against high-fat diet induced male mouse reproductive dysfunction and to explore circadian regulation mechanisms. Male C57BL/6 mice were randomly divided into three groups and fed a normal chow diet (control group, CON), a high-fat diet (HFD group) or a HFD supplemented with fish oil (FO group) for 12 weeks. After 12 weeks of feeding, the body weight and the ratio of perinephric and epididymal fat weight to body weight were significantly higher in the HFD group compared with the CON group. The supplement of fish oil rich in ω-3PUFAs only slightly reduced the HFD-induced obesity but remarkably ameliorated HFD-induced dyslipidemia, sexual hormones disorder, testicle lesions and germ cell apoptosis. Fish oil supplementation restored the expression of steroid synthesis associated genes in HFD fed mouse and flattened the HFD-induced oscillations in circadian genes’ expression. Fish oil supplementation prevented HFD-induced male mouse reproductive dysfunction and modified the rhythmic expression of testosterone synthesis related genes.

## 1. Introduction

Lipid metabolism is closely associated with reproduction. Epidemiological data have revealed that chronic high fat intake is accompanied by a rise of the incidence of metabolic diseases, such as obesity and non-alcoholic fatty liver disease, as well as the morbidity of reproductive dysfunction [1]. The relationship between high fat diet induced metabolic disorder and reproduction has attracted much attention. A number of studies have pointed out that a high-fat diet induces lipid metabolic disorder and leads to a loss of male reproductive function, such as low serum testosterone level, sexual hormones metabolic chaos, reproductive cell apoptosis and poor sperm quality [2,3,4,5,6]. On the other hand, a decreased serum testosterone level aggravates the metabolic syndrome then leads to a vicious loop [7]. Testosterone, the primary androgen synthesized in Leydig cells, plays key roles in the development of male reproductive tissues and spermatogenesis. Several proteins take part in testosterone synthesis; steroidogenic acute regulatory protein (STAR) is in charge of transporting cholesterol to the inner mitochondria membrane and cholesterol side-chain cleavage enzyme (*P450scc*) is the key rate-limited enzyme in testosterone synthesis, catalyzes the oxidative cleavage of the side-chain of cholesterol to form pregnenolone [8,9,10]. Then pregnenolone was transported to smooth endoplasmic reticulum by 3β-hydroxysteroid dehydrogenase (*3β-Hsd*) and reduced by 17β-hydroxysteroid dehydrogenase (*17β-Hsd*) to yield testosterone [10,11]. Some studies have illustrated that high-fat intake and obesity down-regulated the expression of *Star* and *P450scc*, leading to reproductive dysfunction [4]. ω-3 Polyunsaturated fatty acids (ω-3PUFAs) have been found to have the capability to regulate the expression of *Star* and *P450scc* and to improve testosterone synthesis, thereby modifying reproduction [12]. Our previous studies have demonstrated that fish oil rich in ω-3PUFAs can modify cholesterol metabolism and peripheral circadian rhythms [13].

Most mammalian activities, including the sleep–wake cycle, hormone secretion, energy metabolism and immune response, present typical circadian rhythms and are regulated by the body’s internal clock [14,15]. The physiological clock consists of the master clock located in the suprachiasmatic nucleus (SCN) and the sub-clock in the peripheral tissues. Light stimulates the retina and transmits the signal to the SCN to integrate and then deliver to the peripheral clock by the neuroendocrine and autonomic nervous systems [16]. At the molecular level, the core clock genes, including *Bmal1*, *Clock*, *Per1/2*, *Cry1/2*, *Rev-erbα*, *Pparα*, *Sirt1* and *Rorα*, construct a transcriptional/translational feedback loop to control the circadian clock [17]. The rhythmic expression of clock genes is controlled by light and physiological activities such as food intake and they regulate energy metabolism in a nuclear receptors dependent way [18].

The quantity and components of daily food play crucial roles in reproductive rhythms [19]. In normal male rats, mice and humans, testosterone secretions present a circadian rhythm [20,21,22] and a high-fat diet reduces the serum testosterone level and disturbs the 24 h cycle [23]. Meanwhile, a high-fat diet changes the normal peripheral rhythms and exacerbates metabolic disorder [24,25,26,27,28]. In our previous study, we pointed out that fish oil supplementation remarkably improved lipid metabolism and rescued the expression of hepatic clock genes in high-fat diet fed animals [13]. However, whether fish oil can ameliorate high-fat diet-induced male mouse testicle dysfunction and circadian rhythmic chaos remains unclear.

In the current study, we aimed to investigate whether fish oil ameliorates high-fat-diet-induced male sexual hormones metabolic chaos and reproductive disorder in a rhythm-dependent way and to explore the effects of fish oil on the circadian expression of genes involved in testosterone synthesis and testicle function in high-fat diet fed male mice.

## 2. Results

### 2.1. Effect of High-Fat Diet (HFD) and Fish Oil Diet (FO) on the Body Weight, Perinephric Fat and Epididymal Fat Weight and the Plasma Total Cholesterol (TC), Triglyceride (TG) Levels

**Figure 1:**
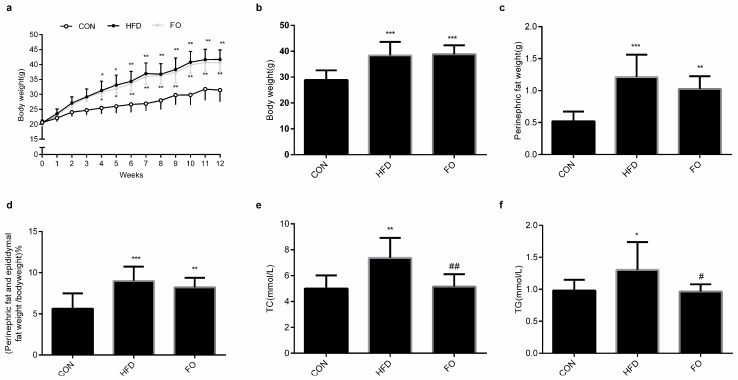
Phenotypic comparison of mice fed the control (CON), high fat diet (HFD), or fish oil (FO) diets for 12 weeks. (**a**) Body weight of mice at 12 weeks. At the end of week 12, the body weight of the three groups (**b**), perinephric fat weight (**c**), the perinephric and epididymal fat weight/body weight ratio (**d**), the plasma total cholesterol (TC) (**e**) and triacylglycerol (TG) (**f**). Data are expressed as mean ± SD (*n* = 10 per group). * *p* < 0.05, ** *p* < 0.01, *** *p* < 0.001 vs. CON group; ^#^ *p* < 0.05, ^##^ *p* < 0.01 vs. HFD group.

After only 4 weeks’ feeding, the body weight of mice in both the high-fat diet (HFD) and fish oil (FO) groups increased significantly compared with the control diet (CON) group (Figure 1a). After 12 weeks’ feeding, the body weight, perinephric fat weight and perinephric and epididymal fat weight/body weight ratios in the HFD and FO groups were significantly higher than those in the CON group (Figure 1b–d). HFD feeding elevated plasma total cholesterol (TC) and the triacylglycerol (TG) level significantly compared with the CON group. Compared with the HFD group, plasma TC and TG concentrations in the FO group decreased significantly (Figure 1e,f).

### 2.2. Effect of HFD and FO on Male Reproductive Organ

**Figure 2:**
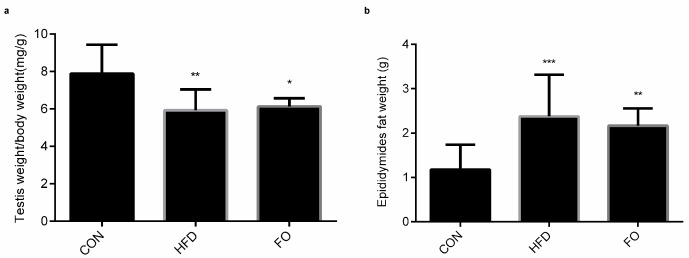
Effect of HFD and FO on male reproductive organs. (**a**) the relative ratio of testes weight/body weight (**b**) the epididymal fat weight. Data were expressed as mean ± SD (*n* = 10 per group). ^*^ *p* < 0.05, ^**^ *p* < 0.01, ^***^ *p* < 0.001 vs. CON group.

The ratio of testes weight/body weight decreased in the HFD and FO groups compared with the CON group (Figure 2a). The epididymal fat weight in the HFD and FO groups were significantly higher than that in the CON group (Figure 2b).

### 2.3. Effect of HFD and FO on Histological Study and Testicular Cell Apoptosis

**Figure 3:**
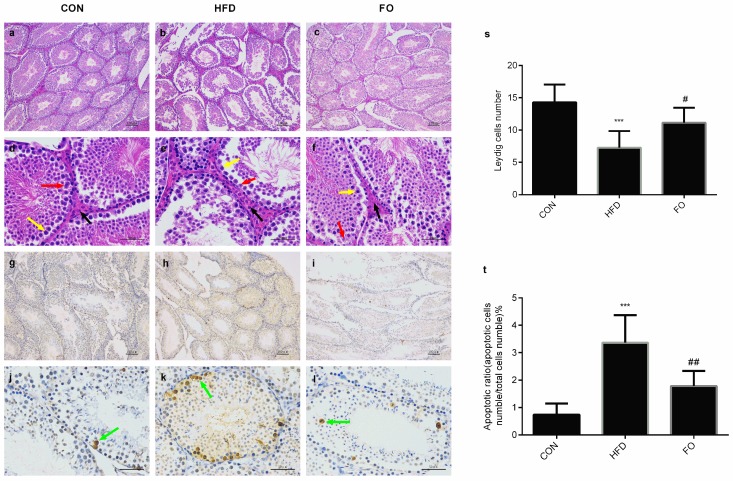
Hematoxylin–eosin (HE) and terminal deoxynucleotidyl transferase-mediated dUTP nick end labeling (TUNEL) staining of testes. Testes tissue sections were stained with HE staining (**a**–**f**) and TUNEL staining (**g**–**l**) respectively and quantitative analysis of the Leydig cells (**s**), apoptosis index (**t**). The representative photographs as follow: (**a**–**c**) H&E staining of the CON, HFD and FO group testes sections (scale bar 100 μm), respectively; (**d**–**f**) H&E staining of the CON, HFD and FO group testes sections (scale bar 50 μm), respectively; (**g**–**i**) TUNEL staining of the CON, HFD and FO group testes sections (scale bar 100 μm), respectively; (**j**–**l**) TUNEL staining of the CON, HFD and FO group testes sections (scale bar 50 μm), respectively. Black, red, yellow, green arrow indicates the Leydig cell, sertoli cell, spermatozoa cell, apoptotic cell, respectively. Data were expressed as mean ± SD (*n* = 10 per group). *** *p* < 0.001 vs. CON group; ^#^ *p*<0.05, ^##^ *p* < 0.01 vs. HFD group.

Histological staining of testicular structure illustrated that, compared with the control group, HFD feeding induced obvious pathological changes in testicular tissue: the bore size of seminiferous tubules increased markedly, the arrangement of spermatogenic cells were disturbed and fewer sperm were observed (Figure 3b–e). These histological changes were improved following supplementation with fish oil (Figure 3c–f). In each field of measurement, the average number of Leydig cells was evaluated adjacent to the seminiferous tubules. As shown in Figure 3s, the average number of Leydig cells was reduced in the HFD group (*p* < 0.001) and which was restored in the FO group. The apoptosis index was found to be higher in the HFD group compared with the control group; after fish oil was added, the apoptosis index went down markedly and no significant difference was seen between the FO group and the CON group (Figure 3g–l,t).

### 2.4. FO Alleviated Testes Testosterone Synthesis Capacity in HFD Mice

**Figure 4:**
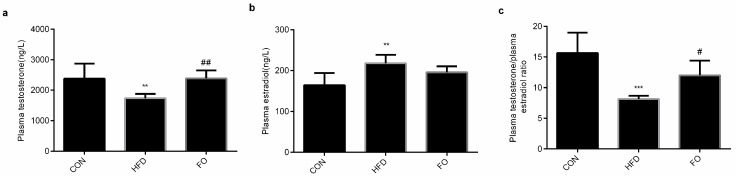
Effects of HFD and FO on plasma testosterone (**a**), estradiol levels (**b**) and the testosterone/estradiol ratio (**c**). Data were expressed as mean ± SD (*n* = 10 per group). ** *p* < 0.01, *** *p* < 0.001 vs. CON group; ^#^ p < 0.05, ^##^ p < 0.01 vs. HFD group.

After 12 weeks’ feeding, plasma testosterone and estradiol levels were measured. The results showed that the plasma testosterone and the ratio of plasma testosterone/estradiol were significantly reduced in the HFD group compared with the CON group, while those changes were restored in the FO group (*p* < 0.01, Figure 4a–c). The plasma estradiol concentrations increased significantly in the HFD group than in the CON group (*p* < 0.01) and slightly reduced with the intake of fish oil (Figure 4b).

**Figure 5:**
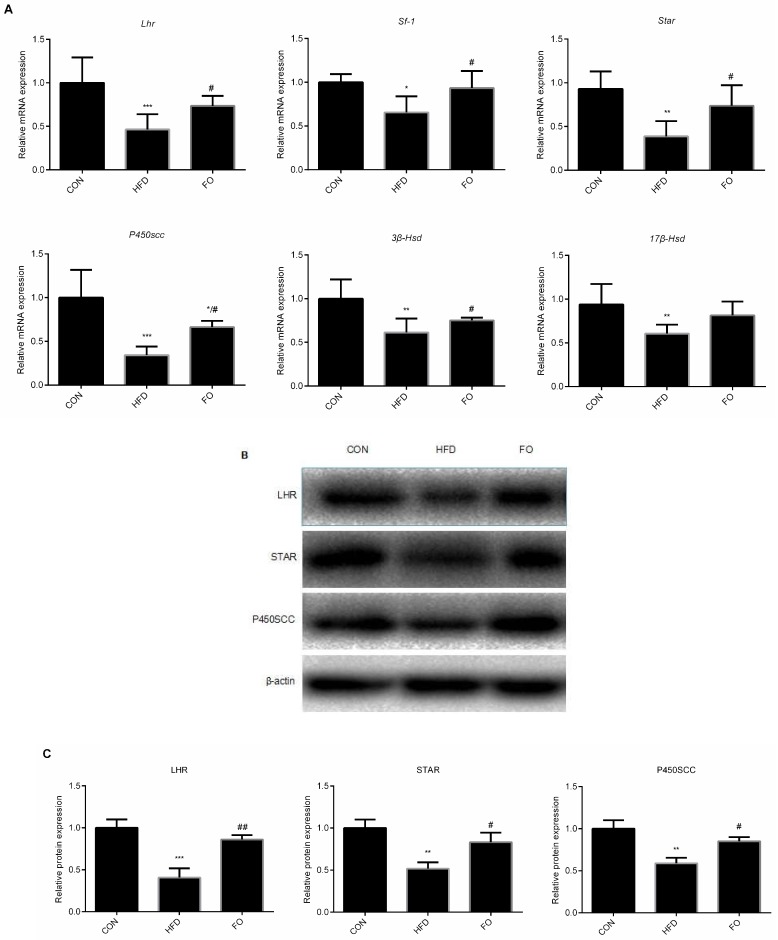
Effect of HFD and FO on the expression of the genes involved in some testosterone synthesis in the testes. (**A**) the mRNA expression of *Lhr*, *Sf-1*, *Star*, *P450scc*, *3β-Hsd*, *17β-Hsd* in the testes. (**B**) Western blot analysis of LHR, STAR, P450SCC expression in the testes. (**C**) The LHR, STAR, P450SCC expression levels were quantitatively analyzed with Image Lab software. Data were expressed as mean ± SD (*n* = 10 per group). * *p* < 0.05, ** *p* < 0.01, *** *p* < 0.001 vs. CON group; ^#^ p < 0.05, ^##^ p < 0.01 vs. HFD group.

After 12 weeks’ feeding, the testicle mRNA levels of *Lhr*, *Star*, *P450scc*, *3β-Hsd*, *17β-Hsd*, *Sf-1* were significantly lower in the HFD group than in the CON group, while the expression of these genes—except *17β-Hsd*—were significantly higher in the FO group than in the HFD group and only the mRNA expression level of *P450scc* was significantly reduced in the FO group compared with the CON group, no significant differences in the other genes were observed between the FO and the CON group (*p* < 0.05, Figure 5A). Additionally, Western blotting analysis indicated that HFD feeding down-regulated the protein levels of LHR, STAR and P450SCC, which were prevented by supplementation with fish oil (Figure 5B,C).

### 2.5. Different Diets Affect the Circadian Rhythm of Testes Testosterone Synthesis

**Figure 6:**
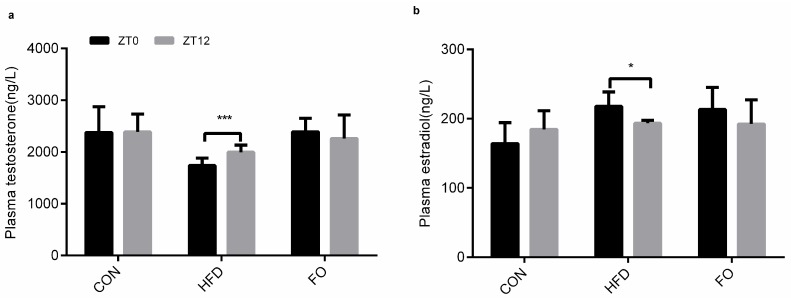
Effect of HFD and FO on the contents of T (**a**) and E2 (**b**) in plasma between morning (zeitgeber time 0, ZT0) and night (zeitgeber time 12, ZT12) variation. Data were expressed as mean ± SD (*n* = 10 per group). * *p* < 0.05, *** *p* < 0.001.

Our data showed that the plasma testosterone and estradiol concentrations had no significant difference between ZT0 and ZT12 in the CON group. Interestingly, significant differences were observed between ZT0 and ZT12 in the HFD group but the differences disappeared in the FO group (Figure 6).

**Figure 7:**
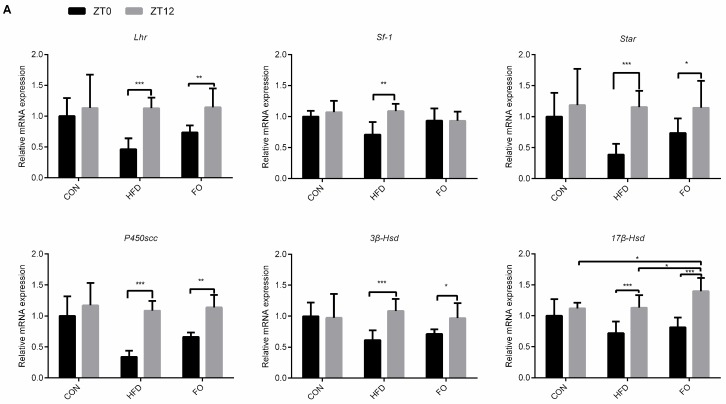

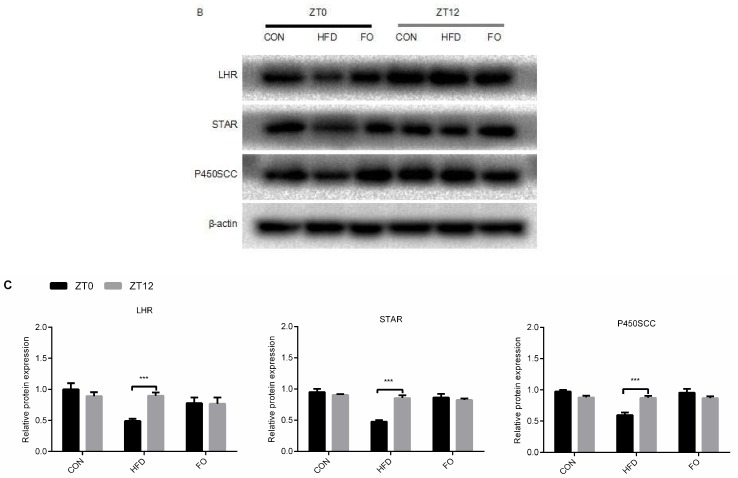
Effect of HFD and FO on the rhythmic expression of some steroidogenic-related genes. (**A**)Rhythmic transcription levels analysis of *Lhr*, *Star*, *P450scc*, *3β-Hsd*, *17β-Hsd*, *Sf-1*genes. (**B**) Western blot analysis of LHR, STAR, P450SCC expression in testes. (**C**) Expression levels of LHR, STAR and P450SCC were quantitatively analyzed with Image Lab software. Data were expressed as mean ± SD (*n* = 10 per group). * *p* < 0.05, ** *p* < 0.01, *** *p* < 0.001.

To explain the circadian oscillations of the sexual hormones in the HFD group, the expression levels of testosterone synthesis genes at ZT0 and ZT12 were checked, the mRNA expression of *Lhr*, *Star*, *P450scc*, *3β-Hsd*, *17β-Hsd* and *Sf-1* and the protein expression of LHR, STAR, P450SCC showed no significant difference between ZT0 and ZT12 in the CON group (Figure 7). The mRNA and protein expression were significantly changed between ZT0 and ZT12 in the HFD group (*p* < 0.05, Figure 7) but this change in the mRNA expression of genes eased in the FO group (*p* < 0.05, Figure 7A)—no change in the protein levels was observed (*p* > 0.05, Figure 7). Additionally, the expression of *17β-Hsd* at ZT12 was significantly increased in the FO group compared with the CON and HFD groups (*p* > 0.05, Figure 7A).

### 2.6. Effect of HFD and FO on Rhythmic Expression of Core Clock Genes

**Figure 8:**
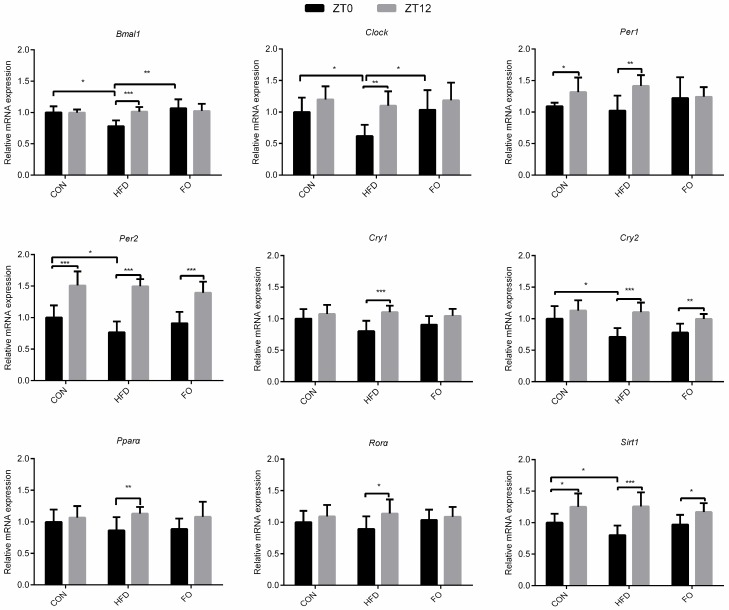
Rhythmic expression of *Bmal1*, *Clock*, *Per1/2*, *Cry1/2*, *Rorα*, *Pparα* and *sirt1* genes. Data were expressed as mean ± SD (*n* = 10 per group). * *p* < 0.05, ** *p* < 0.01, *** *p* < 0.001.

Testes, used for the RT-qPCR assay of core clock genes, were collected at ZT0 and ZT12 from the three groups of mice. The circadian expression of genes was controlled by clock genes. At ZT0, the HFD feeding significantly down-regulated the expression of clock genes (*Bmal1*, *Clock*, *Per2*, *Cry2*, *Sirt1*) (*p* < 0.05) and the expression of *Bmal1*, *Clock* genes was rescued by fish oil feeding. However, no differences were found at ZT12 between the three groups. The rhythmic expression of clock genes between ZT0 and ZT12 were analyzed. The testicle mRNA levels of *Bmal1*, *Clock*, *Cry1*, *Cry2*, *Rorα* and *Pparα* genes at ZT0 and ZT12 showed no remarkable differences in the CON group (*p* > 0.05). In the HFD group, all the examined clock genes presented oscillated expression (*p* < 0.05) and the oscillations of *Bmal1*, *Clock*, *Per1*, *Cry1*, *Rorα and Pparα* genes were abated in the FO group (Figure 8).

## 3. Discussion

Numerous studies have revealed the negative effects of a high-fat diet on male reproduction [2,3,4,5,6]. In the present study, after 12 weeks of high-fat feeding, remarkable features of obesity, including increased body weight, perinephric and epididymal fat weight, hypercholesteremia and hypertriglyceridemia, were identified in mice (Figure 1). However, testicle weight was not increased to accompany the elevation in body weight (Figure 2). The structure of testes in the HFD group was damaged with reduced Leydig cells number and increased apoptotic reproductive cells (Figure 3) and sexual hormones were disordered (Figure 4). A restricted diet with low fat has potential benefits for testicle tissues in mice [29]. A recent study indicated that ω-3PUFAs can also relieve reproductive disorder induced by a high-fat diet [12]. Clinical data illustrated that monounsaturated fatty acid intake is inversely proportional to serum testosterone levels, whereas ω-3PUFAs intake is proportional to serum testosterone levels [30]. In the present study, a high fat diet supplemented with fish oil reduced plasma TC and TG levels compared with the HFD group (Figure 1) and prevented the effect of high-fat on testicular structure (Figure 3). The disturbed plasma testosterone, estradiol levels and testosterone/estradiol ratio were recovered by fish oil supplementation (Figure 4). The results were consistent with Li et. al’s study [12], which suggests that supplementation with fish oil rich in ω-3PUFAs improved sexual hormones synthesis in high-fat induced obese mice.

Testosterone synthesis level is related to the number of Leydig cells in the testes [31]. *Sf-1*, a member of the orphan nuclear receptor family, can stimulate the expression of *Star* and all other genes involved in testicular steroids synthesis [32,33]. *Lhr* is another gene in charge of testosterone synthesis, a study has demonstrated that the expression of *Star*, *P450scc* and *17β-Hsd* were all significantly reduced in *Lhr* knock-out mice compared with wild type [34]. In our current study, the testicular mRNA levels of *Lhr*, *Sf-1*, *Star*, *P450scc*, *3β-Hsd*, *17β-Hsd* and the protein levels of LHR, STAR, P450SCC were all significantly higher in mice testes in the FO group than in the HFD group (Figure 5). These data supported our findings that fish oil supplementation prevented HFD-induced testosterone level reduction. Restored testosterone levels in the FO group may be attributed to an increased total number of Leydig cells and a reduced number of apoptotic cells, which led to an elevated expression of testosterone synthesis related enzymes.

A classic opinion is that the peripheral circadian clock is not presented in normal mice testicles [35,36,37,38]. Zylka et al. identified that *Per1* and *Per3* transcription levels had circadian oscillations and *Per2* was not detected by Northern blotting [39]. However, Alvarez et. al found that these genes had no circadian expression oscillations in testicles using Rnase protection assay and RT-qPCR [36,37,38,40,41]. In the current study, the expression of clock genes such as *Bmal1*, *Clock*, *Cry1/2*, *Pparα* and *Rorα* had no significant differences between ZT0 and ZT12 (*p* > 0.05, Figure 8), which were consistent with previous studies but circadian expression of *Per1* and *Per2* was observed (*p* < 0.05, Figure 8) [39,42]. After 12 weeks of HFD feeding, oscillations in the expression of clock genes between ZT0 and ZT12 were observed, which disappeared for the most studied genes with fish oil supplementation (Figure 8).

Circadian rhythm has been thought to affect reproductive functions. Deficiency of the *Bmal1* gene in mice resulted in disturbed reproduction, low testosterone levels and reduced the expression of *Star* and other testosterone synthesis associated genes [43,44]. In *Bmal1*, *Per2* or *Per1/2* knockout mice, the testicular *Star* expression was significantly inhibited, whereas *Per1* deficiency has no effects on *Star* expression [43]. These findings suggest that clock genes such as *Bmal1* and *Per2* contribute to the regulation of testosterone synthesis. In the present study, the expression of testosterone synthesis-related genes, including *Lhr*, *Sf-1*, *Star*, *P450scc*, *3β-Hsd* and *17β-Hsd*, had no significant difference between ZT0 and ZT12 in the CON group (Figure 7), the findings were consistent with other studies [43]. Interestingly, changes in the expression of these genes between ZT0 and ZT12 were observed in the HFD group (Figure 7), which suggested that the disorder of testosterone synthesis in HFD mice may be partly attributed to circadian rhythm chaos. Fish oil supplementation prevented the normal rhythms from being disrupted.

In conclusion, fish oil rich in ω-3PUFAs can ameliorate the chaos of sexual hormones synthesis in high-fat induced obese male mice by increasing the number of Leydig cells and inhibiting apoptosis in reproductive cells and then stimulating the expression of *Lhr*, *Star* and testosterone synthesis associated enzymes through the adenylyl cyclase–cyclic adenosine monophosphate–protein kinase A (AC–cAMP–PKA) and *Sf-1* pathways. To our knowledge, this is the first finding that high-fat feeding disturbed normal testicular rhythms, disrupted the circadian expression of clock core genes and steroidogenesis-related gene expression in testicles, which may lead to reproductive dysfunction. Furthermore, the protective effects of fish oil against a high-fat diet and the induced disorder of testosterone synthesis, may be partly attributed to the rescue of testicular rhythms.

## 4. Materials and Methods

### 4.1. Animals and Diets

This animal study was conducted according to the Guidelines for the Care and Use of Experimental Animals, and the protocol was approved by Laboratory Animal Ethics Committee of Wuhan Polytechnic University (ID Number: 20160709003) on 9 July 2016. Male C57BL/6 mice (7 weeks old, weighing 18–20 g) were purchased from the Center for Disease Control of Hubei province (Wuhan, China) and were housed in a 12 h day/night cycle with a room temperature of 22 °C ± 1 °C. Mice were randomly allocated to three groups (20 animals per group) and each group was fed one of the following three diets for 12 weeks: a normal chow diet with 10 kcal% fat (CON group), a high fat diet with 42 kcal% fat and 0.5% cholesterol (*w*/*w*) (HFD group), a fish-oil-rich diet with 6.5% fish oil (*w*/*w*) and a total of 42 kcal% fat with 0.5% cholesterol (*w*/*w*) (FO group). Detailed information on these diets is presented in Table 1.

At the end of 12th week of feeding, mice were sacrificed by way of CO_2_ suffocation. Blood was distributed into a heparinized tube (1.5 mL) and centrifuged at 1000× *g* for 15 min at 4 °C and plasma was collected and stored at −80 °C until analysis. Concurrently, the testes, perinephric fat and epididymal fat were rapidly removed, rinsed with 0.9% sodium chloride solution and weighed. Isolated testes were kept at −80 °C or fixed in 10% neutral buffered formalin.

### 4.2. Measurement of Plasma Parameters

Plasma TC (A111-1, Jiancheng Technology, Nanjing, China), TG (A110-1, Jiancheng Technology), estradiol (JYM0379Mo, Wuhan ColorfulGene Biological Technology, Wuhan, China) and testosterone (JYM0373Mo, Wuhan ColorfulGene Biological Technology) were determined using respective kits according to the manufacturer’s instructions.

### 4.3. Histological Studies and TUNEL Assay

Fixed testes were processed for HE staining according to a previously described method [2]. In each group, 30 fields (five fields per mouse, ×400 magnification) in six mice were randomly selected to count Leydig cells and the data mean values for each parameter were calculated. Testicular cell apoptosis was measured using a TUNEL assay kit (11684817910, Roche Applied Science, Basel, Switzerland) according to the instructions provided by the manufacturer. The number of TUNEL-positive cells and total cells were counted in 10 randomly selected fields per slide at magnification 400×.

### 4.4. Real-Time Polymerase Chain Reaction (PCR) Analysis

Total RNAs from mouse testes were extracted using the TRIzol reagent (Takara Bio, Dalian, China). cDNA was synthesized using the primeScript™ RT Reagent Kit (RR037A, Takara Bio). A 20μL amplification reaction consisted of iTaq Universal SYBR Green Supermix (172-5124, Bio-Rad, Hercules, CA, USA) with 300 nM of both reverse and forward primers. All reactions were performed on the RT-qPCR system (CFX96™, Bio-Rad). The thermal cycling conditions were for 2 min at 50 °C and 3 min at 95 °C, followed by 40 repeats at 95 °C for 15 s, 60 °C for 20 s and 72 °C for 30 s. Primer sets used for q-PCR are listed in Table 2.

### 4.5. Western Blot

Testicular samples were homogenized in lysis buffer containing 50 mM Tris-HCl (pH 7.6), 150 mM NaCl, 1 mM EDTA-Na_2_ (pH 7.4), 1% Triton X-100, 1% sodium deoxycholate, 0.1% SDS, 1mM phenylmethylsulfonyl fluoride (PMSF), 10 mM NaF and 1 mM Na_3_VO_3_ and centrifuged at 15,000× *g* for 15 min. Protein concentrations were measured using the bicinchoninic acid (BCA) assay (T9300A, Takara Bio-Rad, Shanghai, China). Equal quantities of protein (20–40 µg) were separated with denaturing sodium dodecyl sulphate 10% polyacrylamide gels under reducing and denaturing conditions and were subsequently transferred onto a polyvinylidene difluoride (PVDF) membrane (16919300, Roche, Mannheim, Germany). Membranes were blocked with 5% (*w*/*v*) bovine serum albumin (BSA) and 0.1% Tween in Tris-buffered saline (pH 7.4) at 37 °C for 2 h, followed by hybridization with an anti-LHR (8449T, ABclonal, Wuhan, China), anti-STAR (8449T, CST, Boston, MA, USA), anti-P450SCC (14217S, CST), anti-β-actin (sc-47778, Santa Cruz Biotechnology, Dallas, TX, USA) primary antibody (1:1000 dilution) at 4 °C overnight. After washing three time in Tris-buffered saline containing 0.1% Tween 20, membranes were hybridized with a horseradish peroxidase-conjugated rabbit immunoglobulin G secondary antibody for 1 h at room temperature. Specific protein expression levels were normalized to β-actin for total protein analyses. The blot was detected with the ChemiDoc™ MP System detection system (Bio-Rad Laboratories, Inc., Hercules, CA, USA). The membranes were scanned and the sum optical density was quantitatively analyzed by Image Lab software (Bio-Rad Laboratories, Inc).

### 4.6. Statistical Analysis

Data are presented as mean ± SD. A one-way ANOVA and Tukey’s post hoc analysis were conducted to establish significant differences. All analyses were performed with Graphpad Prism (Version 6.0, Graphpad Software Inc., San Diego, CA, USA) statistical software. Differences were considered statistically significant at *p* < 0.05.

## Figures and Tables

**Table 1:** Feed formulation, feed energy constitute and the total fatty acids profile of experimental diets.

Feed Formulation (g)	Control Diet (CON)	High-Fat Diet (HFD)	Fish Oil Diet (FO)
casein	141.8	178	178
corn starch	565.692	489	489
dextrin	155	0	0
cream	0	148	110
corn oil	40	60	33
fish oil	0	0	65
fibrin	50	60	60
vitamin premix	45	60	60
TBHQ	0.042	0.042	0.042
choline chloride	2.5	0	0
cholesterin	0	4.63	4.725
total	1000	1000	1000
**Calories (kcal/g)**			
protein	14.2%	14%	14%
carbohydrate	75%	44%	44%
fat	10%	42%	42%
total	3.6	4.5	4.5
cholesterin	0	0.5%	0.5%
**Fatty acid composition**			
∑SFA	15.5%	49.70%	47.76%
∑MFA	32%	32.83%	30.30%
∑n−6	53%	17.14%	10.98%
∑n−3	1%	0.33%	10.96%
∑PUFA	54%	17.47%	21.94%
n−6/n−3	53	52	1.002

**Table 2:** Primer sequences.

Name	Sequence (5′ → 3′)
β-Actin-F	GGAGATTACTGCCCTGGCTCCTA
β-Actin-R	GACTCATCGTACTCCTGCTTGCTG
Bmal1-F	CGTTCACTCAGGACAGACAGATAAG
Bmal1-R	TGTGGCGAAGGTAGGATAGGC
Clock-F	CGTTCACTCAGGACAGACAGATAAG
Clock-R	TGTGGCGAAGGTAGGATAGGC
Per 1-F	CCTCCTCCTACACTGCCTCTTC
Per 1-R	CACCACGCTCTCTGCCTTATTG
Per 2-F	GCCAACACAGACGACAGCATC
Per2-R	TCTCCTGGTCCTCCTTCAACAC
Cry 1-F	GCACCAGAAGGCATCCAGAAG
Cry 1-R	GGACCGAGGCGAGAAGACC
Cry 2-F	TGGCAAGGAGGAGAGACAGAAG
Cry2-R	GAAGAGGCGGCAGGAGAGG
Pparα-F	ATTTCCCTGTTTGTGGCTGC
Pparα-R	CGAAGGTCCACCATTTTTTG
Rorα-F	GTGGCTTCAGGAAAAGGTAAA
Rorα-R	GTCGCACAATGTCTGGGTAT
Sirt1-F	TGGTTCATTTATCAGAGTTGCC
Sirt1-R	CATTGTTGTTTGTTGCTTGGTC
Lhr-F	TTGTCGTCATCTGTGCTTGCTAC
Lhr-R	TTTGAGTTGGTGACAGTGATAAGGG
Sf1-F	GGAGCGGCACACCCTTATTA
Sf1-R	CCAACTTTCCCTTCTTTCACT
Star-F	AACTCACTTGGCTGCTCAGTATTG
Star-R	CAGGTGGTTGGCGAACTCTATC
P450scc-F	TGCTGCGGGCTGAAGTCC
P450scc-R	TGTCTCCTTGATGCTGGCTTTG
3β-Hsd-F	CAAGGTGACAGTGTTGGAAGGAG
3β-Hsd-R	AATGATGGCAGCAGTATGGATGAC
17β-Hsd-F	GTGGTTATGAGCAAGCCCTGAG
17β-Hsd-R	GAAGCGGTTCGTGGAGAAGTAG

## Abbreviations

AC	Adenylyl cyclase
*β-Actin*	Actin, beta
*Bmal1*	Aryl hydrocarbon receptor nuclear translocator-like
BSA	Bovine serum albumin
cAMP	Cyclic adenosine monophosphate
*Clock*	Circadian locomotor output cycles kaput
CON	Control diet
*Cry1/2*	Cryptochrome 1/2
*3* */17β-Hsd*	3β-Hydroxysteroid dehydrogenase
HE	Hematoxylin-eosin
HFD	High fat diet
FO	High fat and fish oil diet
KO	Knockout
Lh	Luteinizing hormone
*Lhr*	Luteinizing hormone receptors
*Per1/2*	Period circadian clock 1/2
PMSF	Phenylmethylsulfonyl fluoride
Pparα	Peroxisome proliferator activated receptor alpha
*P450scc*	Cholesterol side-chain cleavage enzyme
PUFAs	Polyunsaturated fatty acids
PVDF	Polyvinylidene difluoride
*Rorα*	RAR-related orphan receptor alpha
*Sf-1*	Steroidogenic factor 1
*Sirt1*	Sirtuin 1
*Star*	Steroidogenic acute regulatory protein
TUNEL	Terminal deoxynucleotidyl transferase-mediated dUTP nick end labeling
RT-qPCR	Quantitative real-time PCR
TC	Total cholesterol
TG	Triacylglycerol
ZT	Zeitgeber time
